# Sonoanatomy and Stepwise/Systematic Ultrasound Examination of the Extrinsic/Intrinsic Wrist Ligaments

**DOI:** 10.3390/diagnostics11101834

**Published:** 2021-10-04

**Authors:** Jia-Chi Wang, Wei-Ting Wu, Ke-Vin Chang, Lan-Rong Chen, Yuko Nakashima, Levent Özçakar

**Affiliations:** 1 Department of Physical Medicine and Rehabilitation, Taipei Veterans General Hospital, Taipei 112201, Taiwan; jcwang0726@gmail.com; 2 School of Medicine, National Yang Ming Chiao Tung University, Taipei 112202, Taiwan; 3 Department of Physical Medicine and Rehabilitation, National Taiwan University Hospital, Bei-Hu Branch, Taipei 10845, Taiwan; wwtaustin@yahoo.com.tw (W.-T.W.); lchen@livemail.tw (L.-R.C.); 4 Department of Physical Medicine and Rehabilitation, National Taiwan University College of Medicine, Taipei 10048, Taiwan; 5 Center for Regional Anesthesia and Pain Medicine, Wang-Fang Hospital, Taipei Medical University, Taipei 11600, Taiwan; 6 Collaborative Research Laboratory of Musculoskeletal Ultrasound in Medicine, Hiroshima University, Hiroshima 734-8551, Japan; yukonaka@hiroshima-u.ac.jp; 7 Department of Physical and Rehabilitation Medicine, Hacettepe University Medical School, Ankara 06100, Turkey; lozcakar@yahoo.com

**Keywords:** sonography, dynamic imaging, carpal, bone, sprain

## Abstract

Ultrasound has emerged as the most useful imaging tool for investigating wrist and hand disorders, with several published ultrasound protocols having demonstrated their practicality in scanning the wrist tendons and nerves. However, ligaments of the wrist are networked in a complex manner, deterring sonographers from examining them with an organized strategy. Furthermore, because of the non-parallel alignment between the radiocarpal, mid-carpal, and carpal–metacarpal joints, precise recognition of the carpal bones is challenging, although ultrasound is paramount for visualizing the wrist ligaments. In this regard, the current article for point of view aims to elaborate sonoanatomy of the carpal bones and to present a stepwise systematic approach for navigating the extrinsic and intrinsic wrist ligaments.

## 1. Introduction

High-resolution ultrasound (US) has been used as a first-line tool for imaging wrist [1,2,3] and hand [4,5] disorders in recent years. Unlike radiography and computed tomography, it excels in delineating soft tissue pathologies and does not involve radiation exposure [6]. Although magnetic resonance imaging has long been considered the gold standard for the evaluation of musculoskeletal injuries, its accessibility, portability, and capability for dynamic assessment are not comparable with those of US [7]. Several previously published US protocols have shown their practicality in scanning wrist tendons [8], nerves [9], and cartilages [10].

The extrinsic and intrinsic wrist ligaments cross-link the radius, ulna, and carpal bones [11], providing stability during motion and stress conditions. Previous reviews [12,13] have discussed the different methods to scan the wrist ligaments, revealing their complexity and deterring sonographers from examining them in an organized manner. Furthermore, because of the non-parallel alignment between the radiocarpal, mid-carpal, and carpal–metacarpal joints [14], precise recognition of the carpal bones is extremely challenging; nevertheless, it is crucial for visualizing wrist ligaments. In this regard, the current article for point of view aims to elaborate the sonoanatomy of the carpal bones and present a stepwise systematic approach to navigate the extrinsic and intrinsic wrist ligaments. The sonographic images presented in this article were obtained using a 3–19 MHz high-frequency linear transducer (X-Cube 90, Alpioion Medical Systems Co. Ltd., Anyang, Korea).

## 2. Sonoanatomy of Carpal Bones

The carpal bones are arranged in proximal and distal rows [15]. The former includes the scaphoid, lunate, triquetrum, and pisiform, while the latter harbors the trapezium, trapezoid, capitate, and hamate. The two rows are not aligned as two parallel straight lines; instead, the joint line between the proximal and distal rows is a semilunar curve shape. Among the eight bones, the pisiform is situated on the volar surface of the triquetrum and is not in the same plane as the other carpal bones. The wrist joint consists of the proximal row of the carpal bones and the distal radius and ulna.

**Figure 1:**
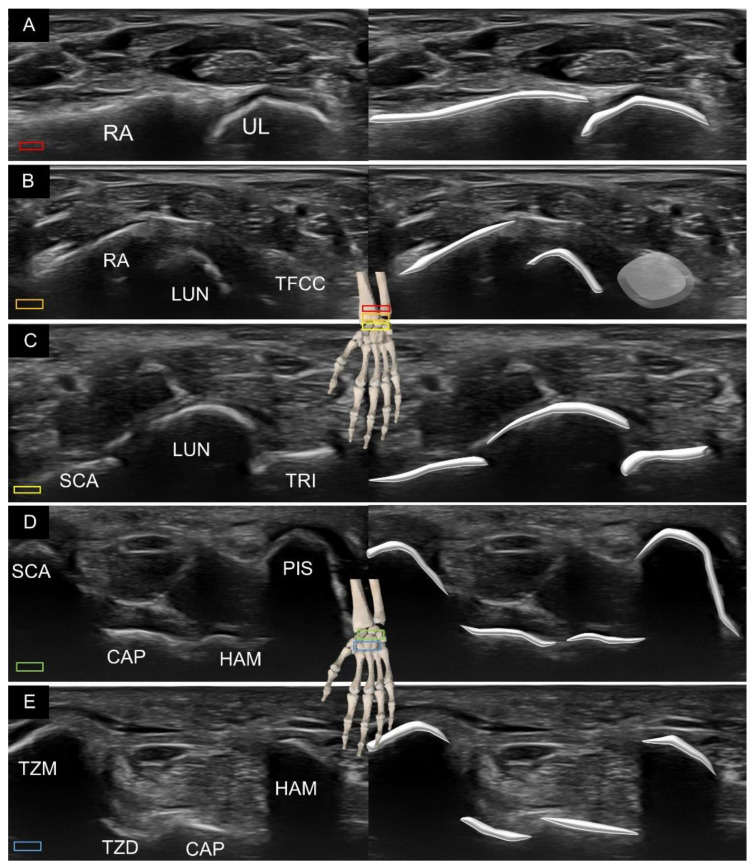
Ultrasound imaging and schematic drawing of the volar wrist in the short axis at the level of the distal radioulnar joint (**A**), triangular fibrocartilage complex (**B**), proximal carpal row (**C**), carpal tunnel inlet (**D**) and carpal tunnel outlet (**E**). RA, radius; UL, ulna; LUN, lunate; TFCC, triangular fibrocartilage complex; SCA, scaphoid; TRI, triquetrum; CAP, capitate; HAM, hamate; TZM, trapezium; TZD, trapezoid. The colored squares are used to indicate the location of the transducer.

When scanning the carpal bones from the palmar aspect, the transducer is placed distal to the wrist crease in the short axis of the forearm on top of the distal radioulnar joint (Figure 1A). When moving the transducer toward the fingers, the first carpal bone seen in the middle of the screen is the lunate. From the radial to ulnar aspect, the radius, lunate, and triangular fibrocartilage can be identified sequentially (Figure 1B).

Relocating the transducer more distally, the scaphoid is visualized at the most radial aspect, whereas the triquetrum is recognized at the ulnar aspect (Figure 1C). Owing to the concavity of the joint line between the proximal and distal carpal rows, the lunate gradually disappears. Instead, if the transducer is bridged between the scaphoid tubercle and pisiform, the capitate and hamate can be identified at the floor of the carpal tunnel inlet (Figure 1D).

Further advancing the transducer more distally, the trapezium tubercle and hamate hook can be recognized as the most superficial bony prominence bridging the carpal tunnel outlet. The trapezoid, capitate, and hamate serve as the floor of the carpal tunnel outlet (Figure 1E).

**Figure 2:**
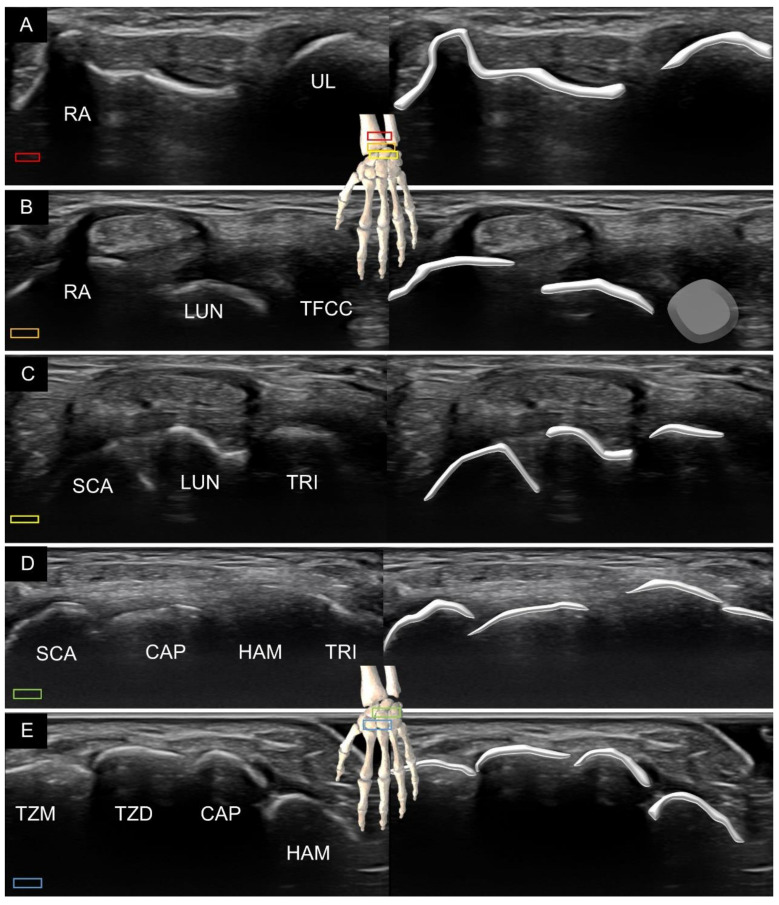
Ultrasound imaging and schematic drawing of the dorsal wrist in the short axis at the level of the distal radioulnar joint (**A**), triangular fibrocartilage complex (**B**), proximal carpal row (**C**), carpal tunnel inlet (**D**) and carpal tunnel outlet (**E**). RA, radius; UL, ulna; LUN, lunate; TFCC, triangular fibrocartilage complex; SCA, scaphoid; TRI, triquetrum; CAP, capitate; HAM, hamate; TZM, trapezium; TZD, trapezoid. The colored squares are used to indicate the location of the transducer.

The principle underlying scanning the dorsal aspect of the carpal bones along the short axis of the forearm is similar to that of the volar side. However, the dorsal surfaces of the eight carpal bones are smoother than their volar surfaces (Figure 2). There are few bony prominences on the dorsal side of the hand. Therefore, correct identification of the carpal bones relies heavily on their reciprocal relationship with the adjacent bones.

**Figure 3:**
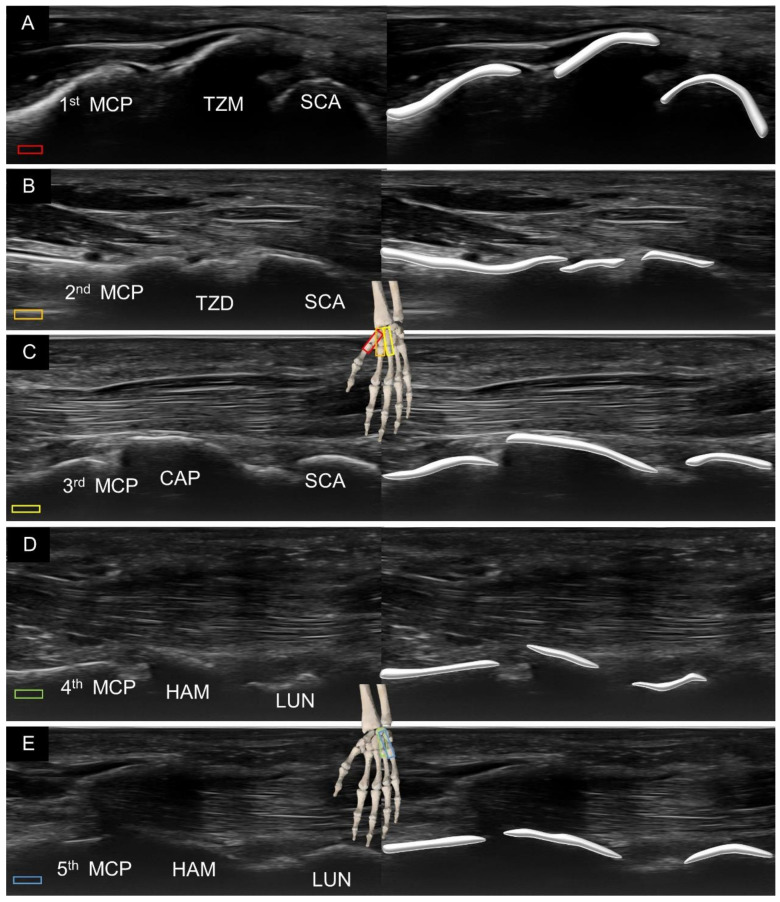
Ultrasound imaging and schematic drawing of the volar wrist in the long axis along the first (**A**), second (**B**), third (**C**), fourth (**D**) and fifth (**E**) metacarpal bones. MCP, metacarpal; TZM, trapezium; SCA, scaphoid; TZD, trapezoid; CAP, capitate; SCA, scaphoid; HAM, hamate; LUN, lunate. The colored squares are used to indicate the location of the transducer.

**Figure 4:**
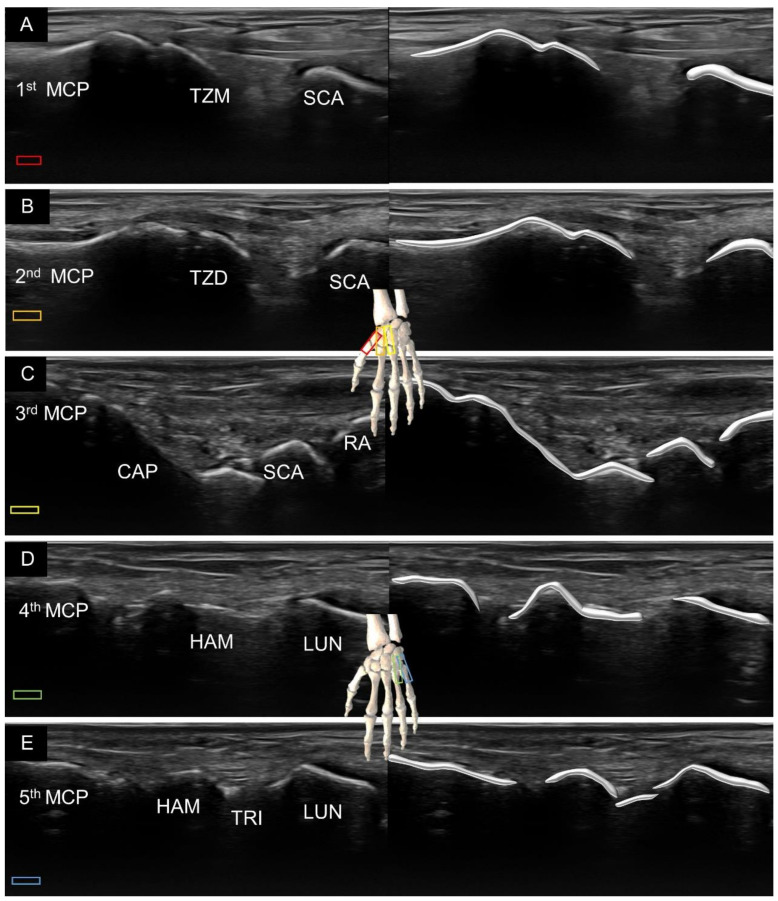
Ultrasound imaging and schematic drawing of the dorsal wrist in the long axis along the first (**A**), second (**B**), third (**C**), fourth (**D**) and fifth (**E**) metacarpal bones. RA, radius; MCP, metacarpal; TZM, trapezium; SCA, scaphoid; TZD, trapezoid; CAP, capitate; SCA, scaphoid; HAM, hamate; LUN, lunate; TRI, triquetrum. The colored squares are used to indicate the location of the transducer.

In this sense, we would like to introduce an alternative method of visualization using the view along the long axis of the finger, either from the volar (Figure 3) or dorsal (Figure 4) aspects of the hand. Placing the transducer along the thumb, the first metacarpal bone, trapezium, and scaphoid can be visualized sequentially. Likewise, along the index finger, the second metacarpal bone, trapezoid, and scaphoid can be identified. Along the middle finger, the third metacarpal bone, capitate, and scaphoid (or lunate) are observed. Along the ring or little finger, the fourth or fifth metacarpal bone, hamate, and lunate are shown.

## 3. Sonoanatomy of Volar Extrinsic Carpal Ligaments

**Figure 5:**
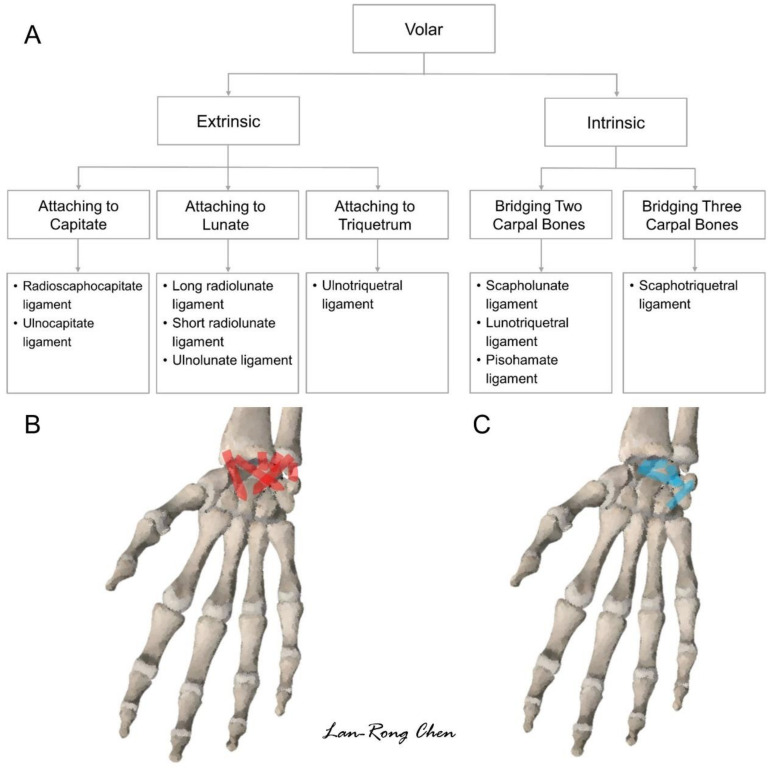
Scanning protocol (**A**) and illustration for the volar extrinsic (**B**) and intrinsic (**C**) wrist ligaments.

Extrinsic carpal ligaments are defined as those connecting the bones of the distal forearm (radius or ulna) with carpal bones [16]. In the volar aspect, several extrinsic carpal ligaments have attachments over the capitate and lunate, which approach the midline of the carpal rows. Ligaments attached to the capitate constitute a greater arc, whereas those that adhere to (or pass over) the lunate form a lessor arc [13]. Both the greater and lesser arcs are crucial for maintaining the stability of the wrist during dorsiflexion. The protocol proposed for scanning the volar wrist ligaments is shown in Figure 5. The positions of the transducer on the real volar wrist are shown in Figures S1 and S2.

### 3.1. Attachment to the Capitate

**Figure 6:**
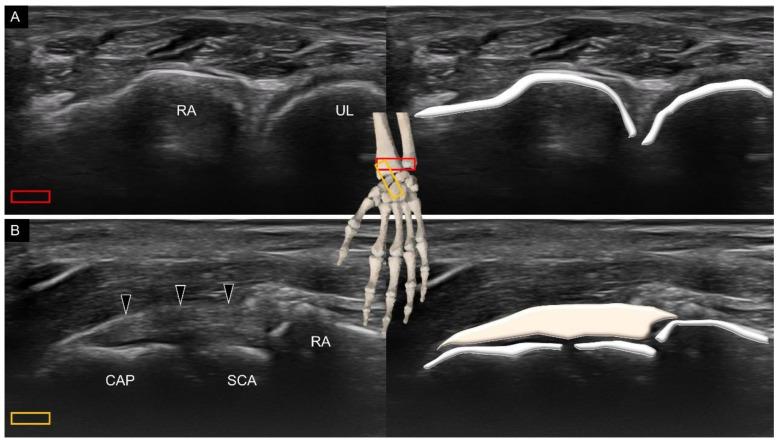
Ultrasound imaging and schematic drawing of the distal radioulnar joint (**A**) and radioscaphocapitate ligament (**B**). RA, radius; UL, ulna; CAP, capitate; SCA, scaphoid; radioscaphocapitate ligament (black arrowheads). The colored squares are used to indicate the location of the transducer.

Two volar extrinsic carpal ligaments have attachment over the capitate, that is, the radioscaphocapitate and ulnocapitate ligaments. To scan the former, the transducer is first placed over the distal radioulnar joint (Figure 6A). Subsequently, it is moved toward the radial aspect to locate its center at the midpoint of the distal radius. The transducer is further rotated 90° along the long axis of the forearm and moved more distally to locate the proximal scaphoid in the middle of the screen. The distal portion of the transducer is pivoted 30° to the midline to visualize the capitate. The radioscaphocapitate ligament spans over the radial styloid process, scaphoid, and capitate (Figure 6B).

**Figure 7:**
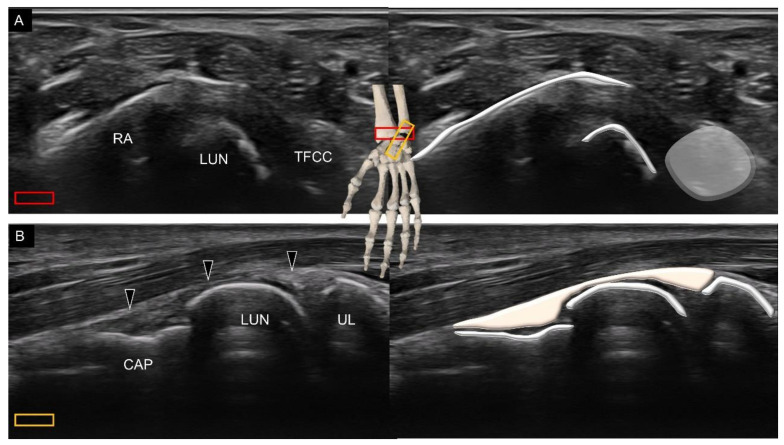
Ultrasound imaging and schematic drawing of the level slightly distal to the radioulnar joint (**A**) and the ulnocapitate ligament (**B**). RA, radius; UL, ulna; CAP, capitate; TFCC, triangular fibrocartilage complex; ulnocapitate ligament (black arrowheads). The colored squares are used to indicate the location of the transducer.

To scan the ulnocapitate ligament, the transducer is placed slightly distal to the distal radioulnar joint (on top of the triangular fibrocartilage complex) in the transverse plane, locating the lunate at the midpoint of the screen (Figure 7A). The radial end of the transducer is pivoted at 60° toward the second finger to demonstrate the capitate. The ulnocapitate ligament spans over the distal ulna, lunate, and capitate (Figure 7B).

### 3.2. Attachment to the Lunate

**Figure 8:**
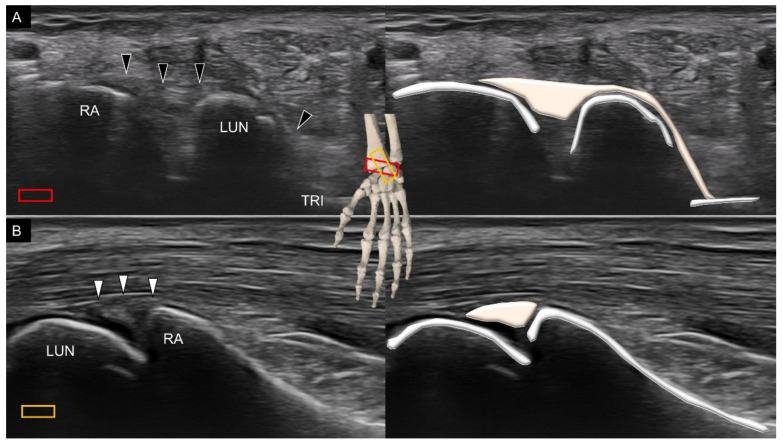
Ultrasound imaging and schematic drawing of the long (**A**) and short (**B**) radiolunate ligaments. RA, radius; LUN, lunate; TRI, triquetrum; long radiolunate ligament (black arrowheads); short radiolunate ligaments (white arrowheads). The colored squares are used to indicate the location of the transducer.

Three volar extrinsic carpal ligaments, the long radiolunate, short radiolunate, and ulnolunate ligaments, have attachment over the lunate. To scan the long radiolunate ligament, the transducer is placed in the transverse plane over the distal radius. Later, the ulnar end is rotated 20° with its radial end as the rotation center to visualize the lunate (Figure 8A). The long radiolunate ligament spans over the distal radius and lunate [17]. As some fibers can be seen extending to the triquetrum, the long radiolunate ligament is also called the radiolunate triquetrum ligament. Moreover, the long radiolunate ligament runs almost parallel to the radioscaphocapitate ligament, with the proximal attachment of the former ligament at the ulnar aspect of the latter. To visualize the short radiolunate ligament, the center of the transducer is placed over the ulnar aspect of the distal radius in the transverse plane. Later, the transducer is rotated to align with the middle finger to visualize the lunate. The short radiolunate ligament is seen as a bridge between the radius and lunate (Figure 8B). Notably, the axis of the short radiolunate ligament is almost vertical to that of the long ligament.

**Figure 9:**
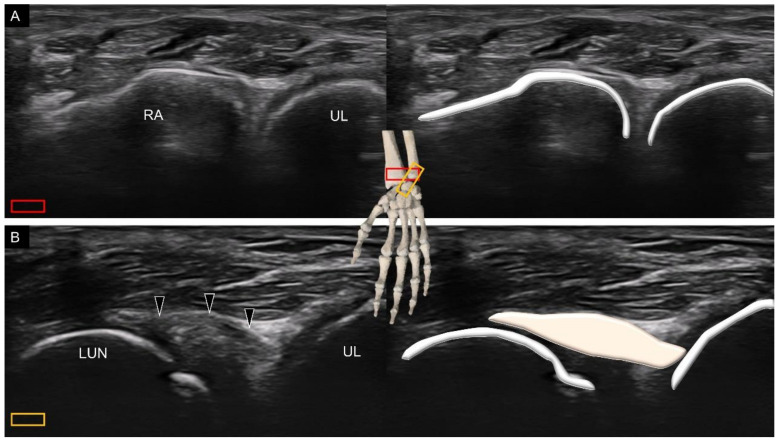
Ultrasound imaging and schematic drawing of the distal radioulnar joint (**A**) and ulnolunate ligament (**B**). UL, ulna; LUN, lunate; ulnolunate ligament (black arrowheads). The colored squares are used to indicate the location of the transducer.

Likewise, the transducer is first placed over the distal radioulnar joint in the transverse plane (Figure 9A), and its radial end is subsequently pivoted toward the index finger to see the lunate with the ulnar end fixed on the ulnar styloid. The ulnolunate ligament is seen bridging the ulna and lunate (Figure 9B).

### 3.3. Attachment to the Triquetrum

**Figure 10:**
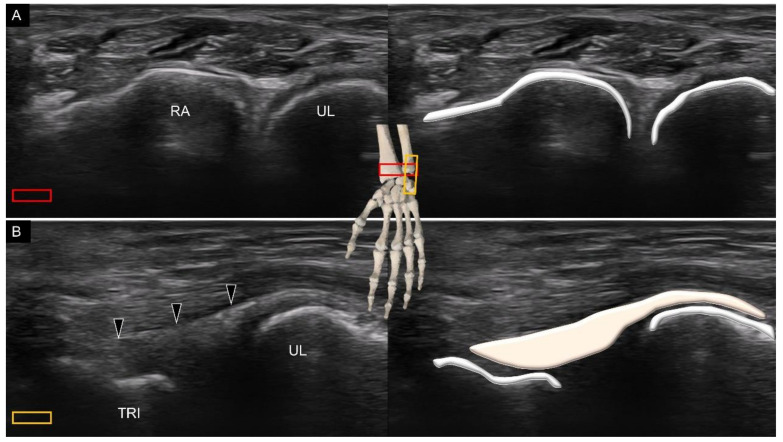
Ultrasound imaging and schematic drawing of the distal radioulnar joint (**A**) and ulnotriquetral ligament (**B**). UL, ulna; TRI, triquetrum; ulnotriquetral ligament (black arrowheads). The colored squares are used to indicate the location of the transducer.

The major extrinsic carpal ligament, which is attached to the triquetrum, is the ulnotriquetral ligament, which also serves as the ventral wall of the triangular fibrocartilage complex. To visualize it, the transducer is first placed over the distal radioulnar joint, with its footprint resting more on the distal ulna (Figure 10A). The transducer is later rotated 90° to cross the triquetrum at the ulnar aspect of the pisiform, whereby the ulnotriquetral ligament is seen bridging the distal ulna and triquetrum (Figure 10B).

## 4. Sonoanatomy of Volar Intrinsic Carpal Ligaments

**Figure 11:**
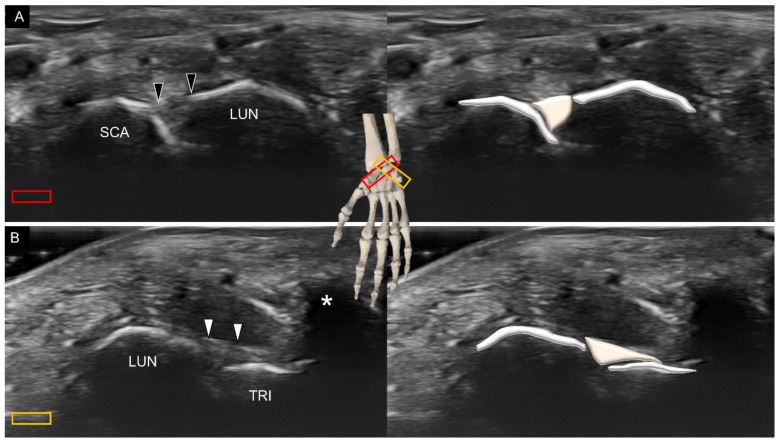
Ultrasound imaging and schematic drawing of the scapholunate (**A**) and lunotriquetral (**B**) ligaments. SCA, scaphoid; LUN, lunate; TRI, triquetrum; scapholunate ligament (black arrowheads); lunotriquetral ligament (white arrowheads); shadow of the pisiform (white asterisk). The colored squares are used to indicate the location of the transducer.

The intrinsic carpal ligaments are located between several adjacent carpal bones, for example, the scapholunate and lunotriquetral ligaments residing over the first carpal row. Therefore, as long as the examiner can identify the carpal bones correctly, it will not be difficult to image the corresponding intrinsic carpal ligaments, such as the volar scapholunate (Figure 11A) and lunotriquetral ligaments (Figure 11B). Furthermore, the intrinsic carpal ligaments are mostly located deeper than the extrinsic ones because they only travel for a short intercarpal distance. When visualizing these short volar intrinsic ligaments, the sonographer should be aware that the scanning plane should be adjusted according to the joint line, instead of parallel to the wrist crease. For example, the radial end of the transducer must be slightly pivoted toward the scaphoid tubercle during examination of the scapholunate ligament. Likewise, the ulnar end of the transducer is required to point toward the pisiform (which is on top of the distal triquetrum) for imaging the lunotriquetral ligament.

**Figure 12:**
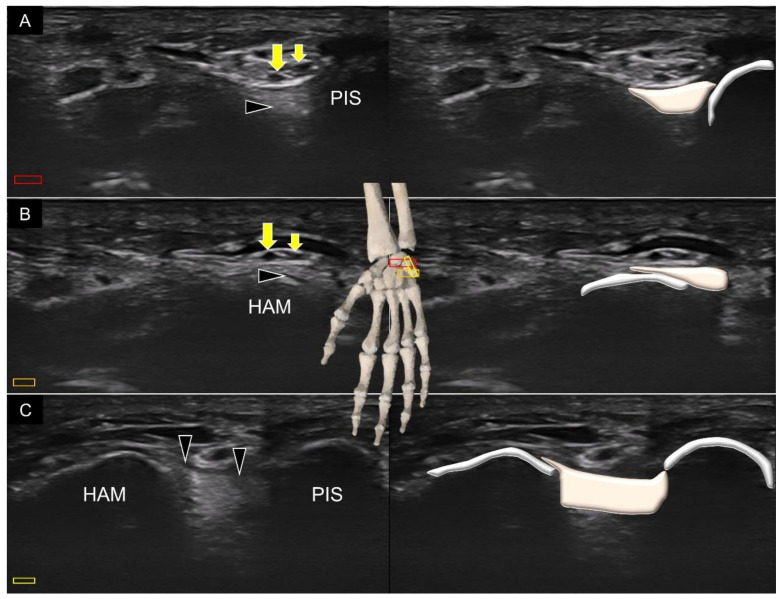
Ultrasound imaging and schematic drawing of the pisohamate ligament at the levels of pisiform (**A**) and hamate (**B**) in the short-axis view, and at its entire length in the long-axis view (**C**). Proper digital nerve of the little finger (small yellow arrows); the common digital nerve of the fourth web space (large yellow arrow); pisohamate ligament (black arrowheads). The colored squares are used to indicate the location of the transducer.

Another important volar intrinsic carpal ligament is the pisohamate ligament [18]. The terminal portion of the ulnar nerve is divided into the deep branch of the ulnar nerve, the proper digital nerve of the little finger, and the common digital nerve of the fourth web space on top of this ligament. The transducer is first placed in the transverse plane at the level of the carpal tunnel inlet, where the pisiform is located at the ulnar side of the screen (Figure 12A). Later, the transducer is moved more distally to confirm the location of the hamate (Figure 12B). Lastly, the transducer is rotated to the sagittal oblique plane to cross the pisiform and hamate to visualize the pisohamate ligament, with the overlying ulnar artery and nerve (Figure 12C).

**Figure 13:**
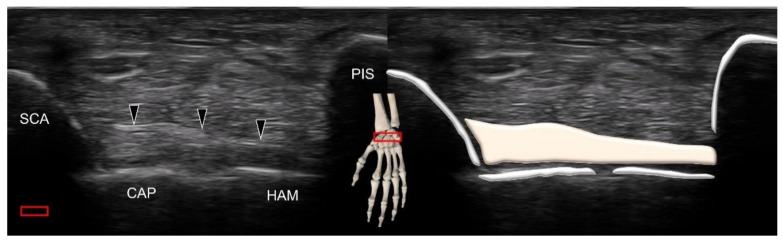
Ultrasound imaging and schematic drawing of the scaphotriquetral ligament (black arrowheads). SCA, scaphoid; CAP, capitate; HAM, hamate; PIS, pisiform. The colored squares are used to indicate the location of the transducer.

The volar intrinsic carpal ligament, which spans several carpal bones, is the scaphotriquetral ligament. Its attachment to the scaphoid is fan shaped [19]. To visualize the ligament, the transducer is placed in the transverse plane near the wrist crease. The ligament can be seen connecting the scaphoid tubercle and triquetrum (underneath the shadow of the pisiform), coursing over the capitate and hamate (Figure 13).

## 5. Sonoanatomy of Dorsal Extrinsic Carpal Ligaments

### Attachment to the Triquetrum

**Figure 14:**
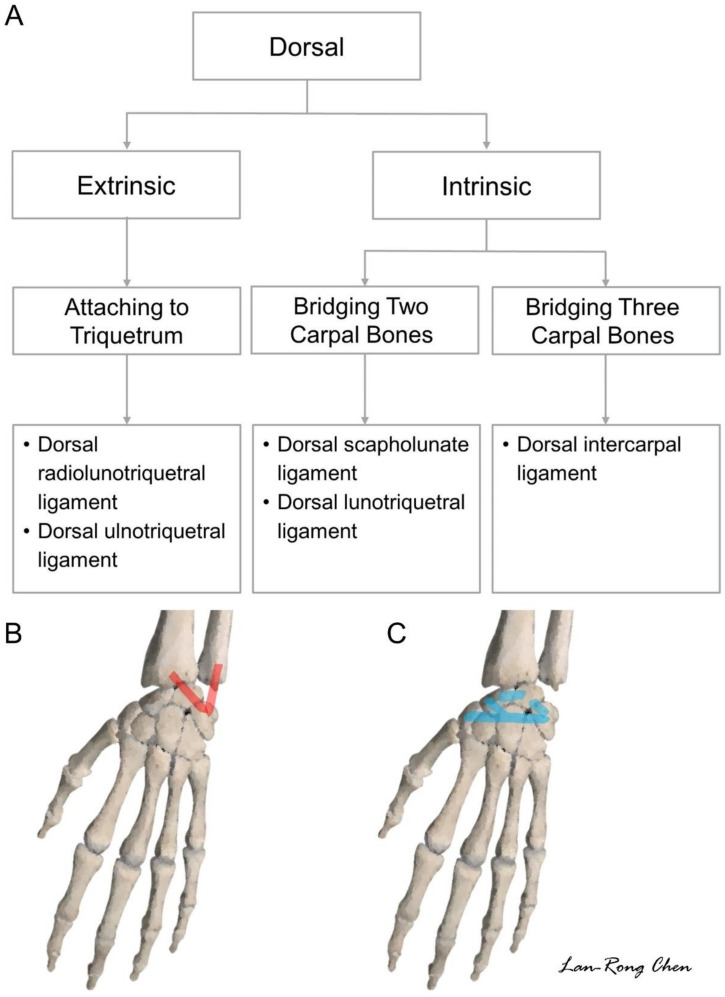
Scanning protocol (**A**) and illustration for the dorsal extrinsic (**B**) and intrinsic (**C**) wrist ligaments.

**Figure 15:**
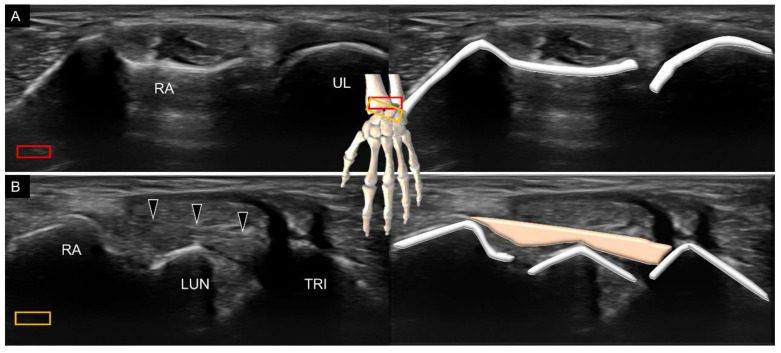
Ultrasound imaging and schematic drawing of the distal radioulnar joint (**A**) and dorsal radiocarpal (**B**) ligaments. RA, radius; UL, ulna; LUN, lunate; TRI, triquetrum; dorsal radiocarpal ligament (black arrowheads). The colored squares are used to indicate the location of the transducer.

The protocol proposed for scanning the dorsal wrist ligaments is shown in Figure 14. The transducer’s positions on the real dorsal wrists are shown in Figures S3 and S4. There are two dorsal extrinsic carpal ligaments attached to the triquetrum, that is, the dorsal radial carpal and dorsal ulnotriquetral ligaments. The former is also called the dorsal radiolunotriquetral ligament. For scanning, the transducer is placed in the transverse plane on the distal radius (Figure 15A). The ulnar end is then pivoted toward the triquetrum. Once the lunate emerges in the middle of the screen, the dorsal radial carpal ligament can be identified spanning over the distal radius, lunate, and triquetrum (Figure 15B).

**Figure 16:**
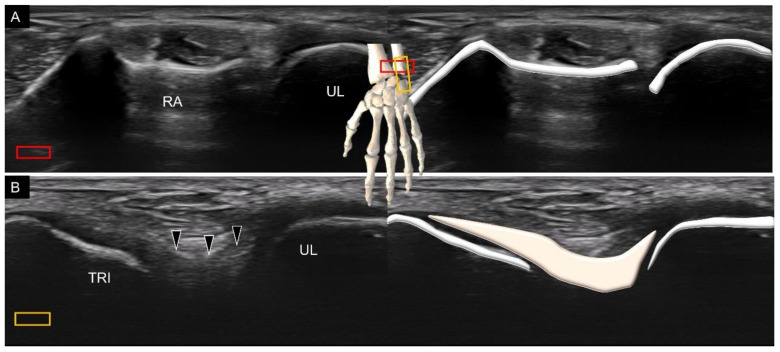
Ultrasound imaging and schematic drawing of the distal radioulnar joint (**A**) and the dorsal ulnotriquetral ligament (**B**). RA, radius; UL, ulna; TRI, triquetrum; dorsal ulnotriquetral ligament (black arrowheads). The colored squares are used to indicate the location of the transducer.

To scan the dorsal ulnotriquetral ligament, the transducer is placed in the transverse plane over the distal radioulnar joint (Figure 16A), with its midpoint resting on top of the distal ulna. The transducer is then rotated 90° until the triquetrum emerges on the screen. Under US imaging, the dorsal ulnotriquetral ligament appears as a thickened portion of the capsule instead of a separate ligament [15] (Figure 16B).

## 6. Sonoanatomy of Dorsal Intrinsic Carpal Ligaments

**Figure 17:**
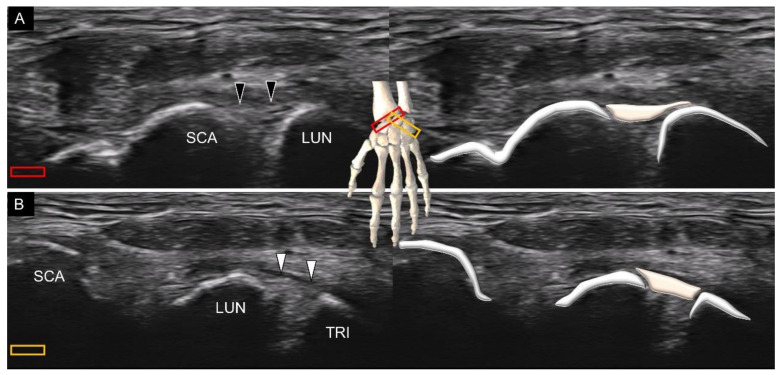
Ultrasound imaging and schematic drawing of the dorsal scapholunate (**A**) and lunotriquetral (**B**) ligaments. SCA, scaphoid; LUN, lunate; TRI, triquetrum; scapholunate ligament (black arrowheads); lunotriquetral ligament (white arrowheads). The colored squares are used to indicate the location of the transducer.

As with the volar wrist, the dorsal intrinsic carpal ligaments can be identified between the adjacent carpal bones, such as the dorsal scapholunate (Figure 17A), and lunotriquetral (Figure 17B) ligaments. The sonographer can take advantage of a towel roll to maintain wrist flexion, and the dorsal intrinsic carpal ligaments can be seen better when stretched.

**Figure 18:**
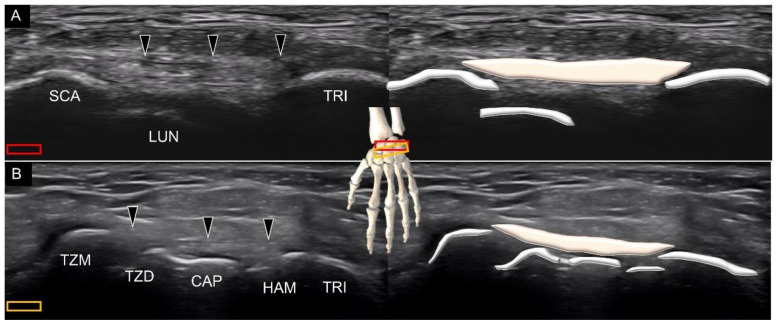
Ultrasound imaging and schematic drawing of the dorsal intercarpal ligament with one radial end on top of the scaphoid (**A**) and the other on top of the trapezium-trapezoid (**B**). SCA, scaphoid; LUN, lunate; TRI, triquetrum; TZM, trapezium; TZD, trapezoid; CAP, capitate; HAM, hamate; dorsal intercarpal ligament (black arrowheads). The colored squares are used to indicate the location of the transducer.

Another dorsal ligament that spans several carpal bones is the dorsal intercarpal ligament [20]. The ulnar end of the transducer is first placed on the triquetrum in the transverse plane. The ligament can be seen originating from the triquetrum, traveling over the lunate with two bony attachments on its radial side. One is the scaphoid (Figure 18A) and the other is the trapezoid–trapezium (Figure 18B). The radial end of the transducer can be slightly pivoted to identify both the radial attachments.

## 7. Sonoanatomy of Wrist Collateral Ligaments

**Figure 19:**
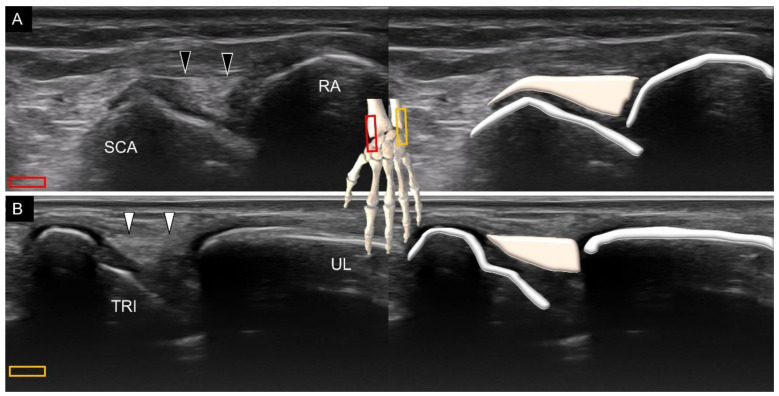
Ultrasound imaging and schematic drawing of the radial (**A**) and ulnar (**B**) collateral ligaments. SCA, scaphoid; RA, radius; TRI, triquetrum; UL, ulna; radial collateral ligament (black arrowheads); ulnar collateral ligament (white arrowheads). The colored squares are used to indicate the location of the transducer.

The collateral ligaments exist in the radial and ulnar aspects of the wrist [21]. The radial collateral ligament connects the distal radius and scaphoid. To visualize this ligament, the transducer is placed in the coronal plane deep to the first extensor compartment of the dorsal wrist. The ligament has a broader origin at the radial aspect and tethers over the scaphoid side (Figure 19A). A slight ulnar deviation of the wrist is helpful for better visualization. The ulnar collateral ligament connects the distal ulna and triquetrum. The transducer is placed in the coronal plane, next to the extensor carpal ulnar tendon. The ligament is seen on top of the triangular fibrocartilage complex (Figure 19B). Slight radial deviation of the wrist facilitates the identification of the ligament.

## 8. Pathology

**Figure 20:**
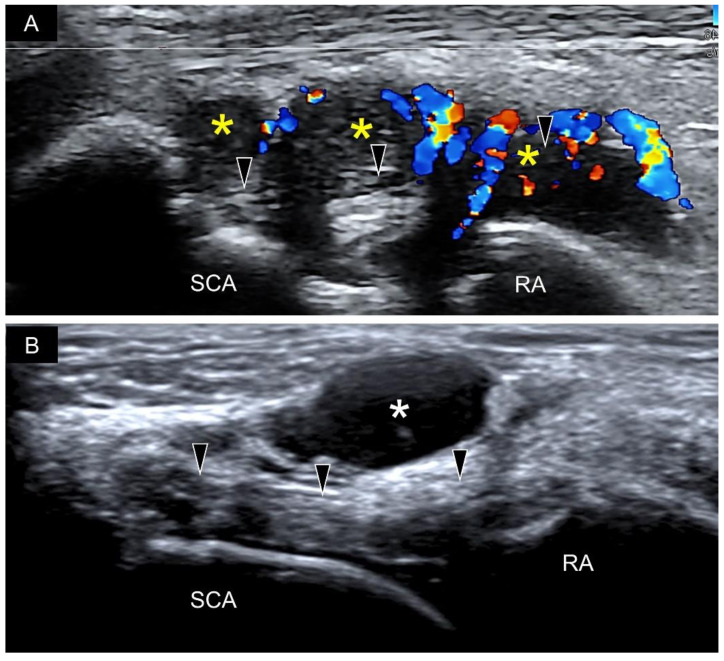
Hypervascularity and synovial hypertrophy (yellow asterisks) are seen beside the radioscaphocapitate ligament (black arrowheads) (**A**). A ganglion cyst (white asterisk) is identified on top of the radioscaphocapitate ligament (black arrowheads) (**B**). SCA, scaphoid; RA, radius.

Disorders of the wrist ligaments can be clearly examined using high-resolution ultrasonography. Compared with computed tomography that is mainly used to evaluate bony abnormalities for musculoskeletal disorders, ultrasonography can delineate connective tissue structures such as the wrist ligaments highlighted in this article. Although MRI excels in the diagnosis of bony and soft tissue disorders, its expense is high and does not allow dynamic examination [6]. Furthermore, compared with MRI that is usually presented in specific cuts (axial, coronal and sagittal), the US transducer can be easily adjusted to align with the entire course of the wrist ligaments, which makes US imaging a suitable tool for exploration of wrist pathologies [1,3,5,22]. A common pathology in clinical settings is sprain injury [23] for which US reveals thickened and hypoechoic (disorganized and wavy) fibers. Power Doppler is helpful in recognizing intra-ligamentous hypervascularity (Figure 20A). When examining the target ligament, the sonographer needs to tighten it by positioning the wrist. The examinee can make a full fist during the inspection of the dorsal ligaments and stretch fingers when the palmar ligaments are being scanned [15]. It is easier to identify the difference between the normal and sprained ligaments under tensile stress. Furthermore, the sprained ligament may be affected at one end with a nearly normal sonographic appearance at the other end. A comparison with the contralateral asymptomatic wrist would be useful at recognizing the trivial abnormalities.

The ganglion cyst (Figure 20B) is another prevalent pathology pertinent to wrist ligaments. Appearing as encapsulated anechoic fluid accumulation, it commonly emerges near the extrinsic or intrinsic wrist carpal ligaments, with a potential link to the underlying joints [24]. Unlike effusion, it is usually incompressible. US-guided aspiration with subsequent corticosteroid injection would be helpful in relieving symptoms if the patient presents with pain and distension over the affected wrist. During aspiration, thicker needles are usually necessary because the chronic dense fluid is unlikely to be drained with thinner needles.

**Figure 21:**
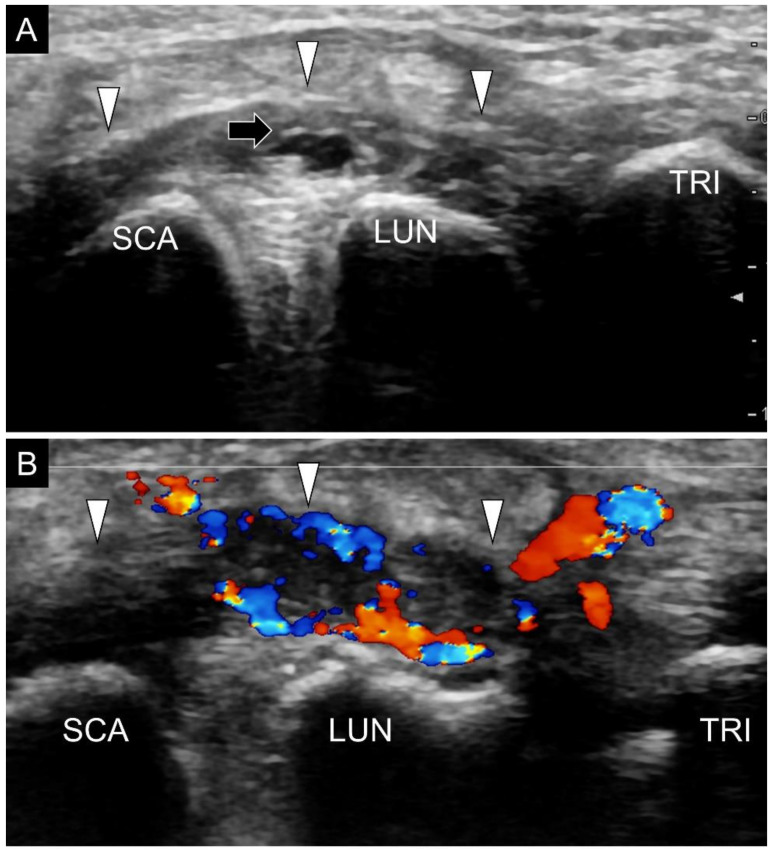
An intra-ligamentous tear (black arrow) (**A**) and peri-ligamentous hypervascularity (**B**) are observed over the dorsal radiocarpal ligament (white arrowheads). SCA, scaphoid; LUN, lunate; TRI, triquetrum.

Wrist ligament tears usually occur after traumatic injury (Figure 21). The absence of ligamentous fibers with effusion filling the gap usually indicates a complete tear [25]. In contrast, thinning of the ligament with intra-ligamentous hypoechogenicity denotes an incomplete tear. Bony chips identified inside the joint space indicate an antecedent avulsion injury. While widening of the joint space during the dynamic stress test is an indirect sign of a ligament tear, clinicians should also keep in mind that ligament problems can also cause impingement [26].

## 9. Strength and Limitation of the Present Article

The strength of our article can be divided into the following three points. First, the wrist ligaments are examined in a systematic method, based on the bony origins (extrinsic vs. intrinsic) and body surfaces (volar vs. dorsal). Second, a stepwise approach is introduced, starting from correct recognition of the carpal bone, and ending at precise alignment of the scanned ligaments. Third, two flow diagrams are attached, enabling the examiners to incorporate the protocol as part of scanning routine. The limitation of the present paper is that comparative MRI images in relation to US images of the same ligaments are not given, which can be considered to incorporate in the future project.

## 10. Conclusions

A stepwise systematic approach is crucial for the thorough examination of the extrinsic and intrinsic wrist carpal ligaments. Familiarization with the arrangement of the carpal bones facilitates the precise recognition of these ligaments. The examiners should be aware of non-parallel alignment between the radiocarpal, mid-carpal, and carpal–metacarpal joint. The diagnosis of pathologies should be based on multiple views of the same ligaments and the comparison with the contralateral asymptomatic side. In addition to static scanning, sonographers should take advantage of dynamic stress tests to identify the presence of joint instability associated with ligamentous injury. Finally, the imaging algorithms proposed in this article are highly recommended during routine daily US imaging to avoid misdiagnosis of relevant wrist pathologies.

## Supplementary Materials

The following are available online at https://www.mdpi.com/article/10.3390/diagnostics11101834/s1, Figure S1: The position of the transducer for visualizing the volar extrinsic carpal ligament, Figure S2: The position of the transducer for visualizing the volar intrinsic carpal ligament, Figure S3: The position of the transducer for visualizing the dorsal extrinsic carpal ligament, Figure S4: The position of the transducer for visualizing the dorsal intrinsic carpal ligament.
