# Supplementary material for: Sonoanatomy and Stepwise/Systematic Ultrasound Examination of the Extrinsic/Intrinsic Wrist Ligaments
Source: Diagnostics (Basel). 2021 Oct 4;11(10):1834. doi: 10.3390/diagnostics11101834 (PMC8534780; doi:10.3390/diagnostics11101834)
Supplement: Supplementary file 1 [file diagnostics-11-01834-s001.zip › diagnostics-1386109-supplementary.pdf]

Supplementary Figures

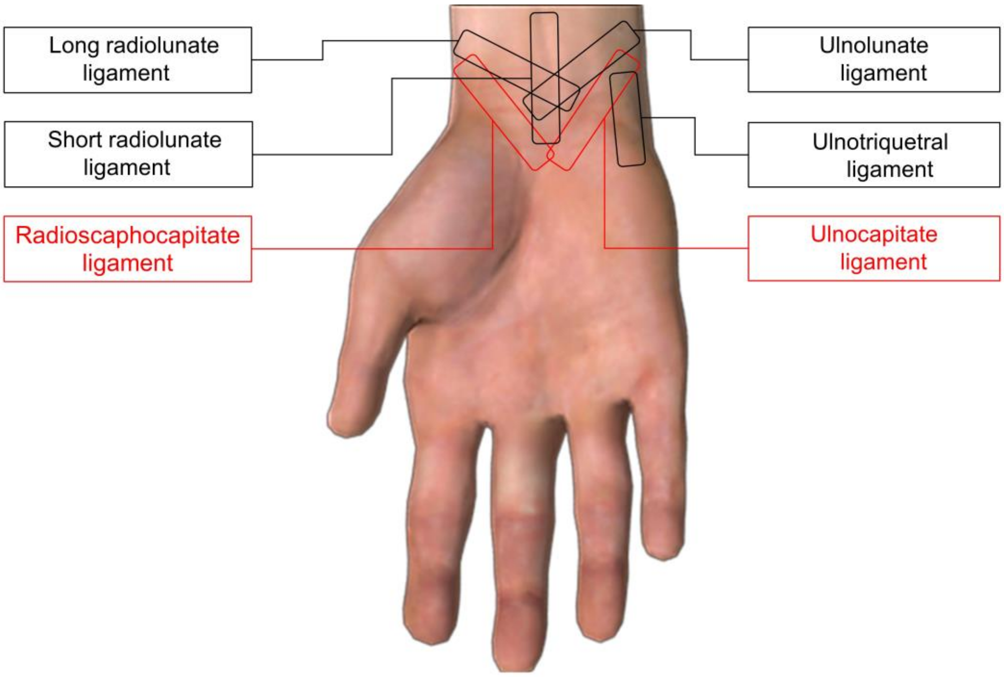

Figure S1. The position of the transducer for visualizing the volar extrinsic carpal ligament.

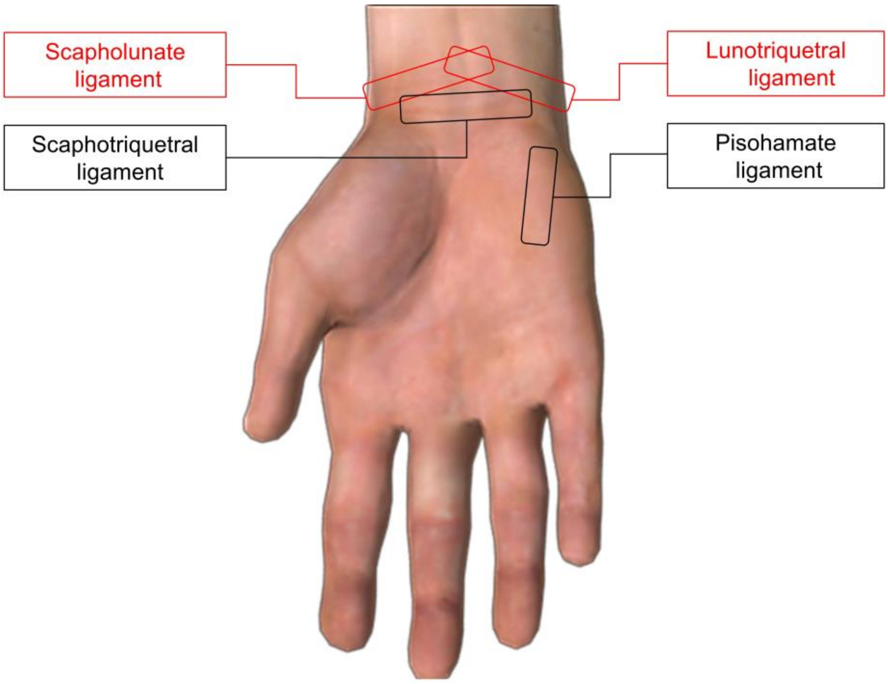

Figure S2. The position of the transducer for visualizing the volar intrinsic carpal ligament.

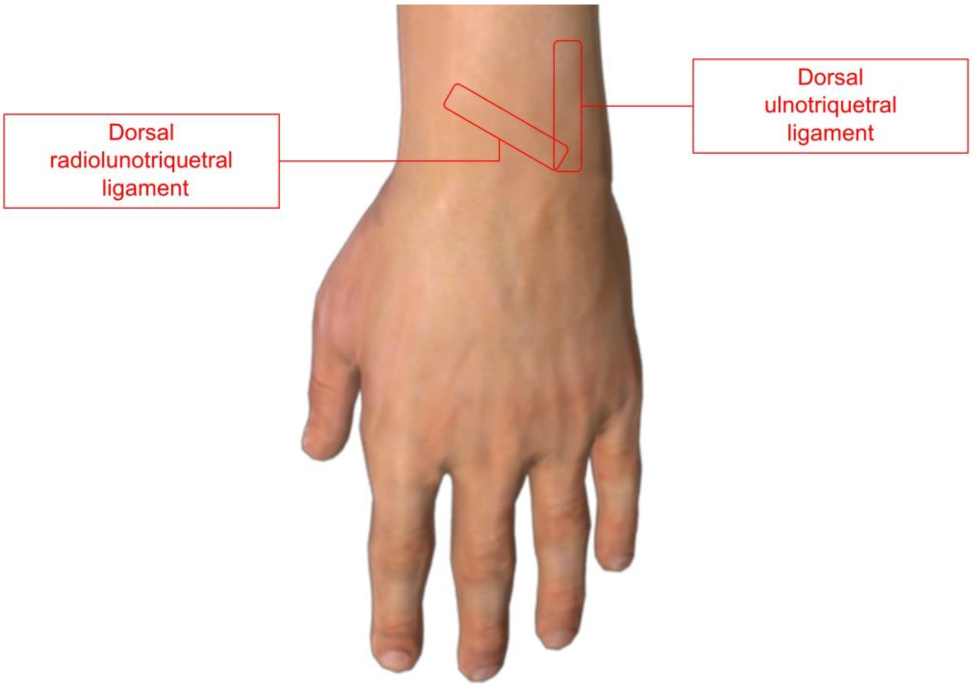

Figure S3. The position of the transducer for visualizing the dorsal extrinsic carpal ligament.

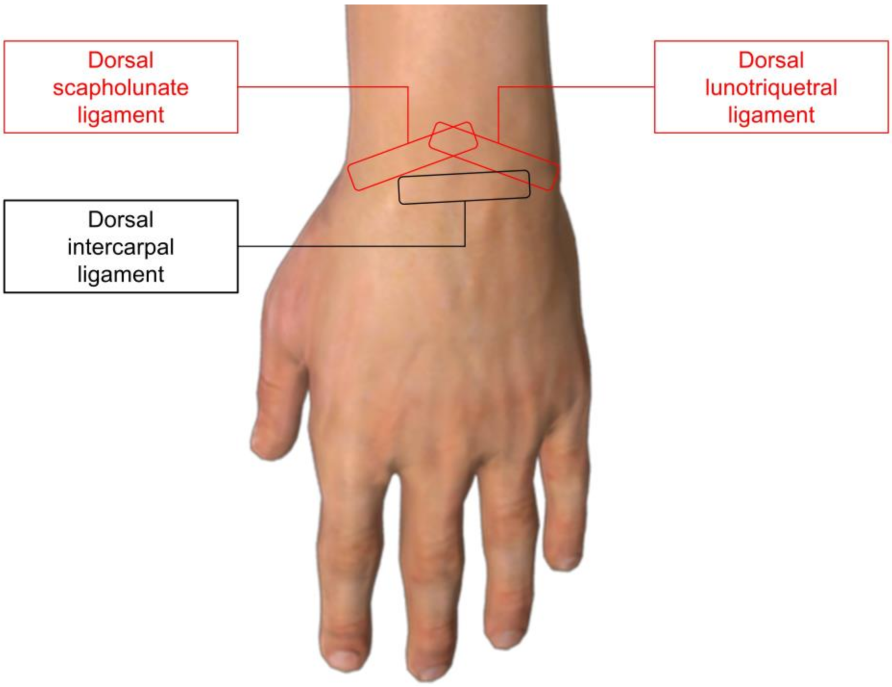

Figure S4. The position of the transducer for visualizing the dorsal intrinsic carpal ligament.
